# Effect of pasteurization on delayed kimchi ripening and regression analysis for shelf life estimation of kimchi

**DOI:** 10.1002/fsn3.915

**Published:** 2019-01-28

**Authors:** Hyun‐Gyu Lee, Suyeon Jeong, Ji Yeong Park, SeungRan Yoo

**Affiliations:** ^1^ World Institute of Kimchi Gwangju Korea; ^2^ Division of Applied Life Science (BK21 Plus) Gyeongsang National University Jinju Korea

**Keywords:** kimchi, lactic acid bacteria, microbial communities, pasteurization, regression analysis, shelf life

## Abstract

Pasteurization‐mediated delayed kimchi ripening and regression analysis for shelf life estimation were investigated. Various initial kimchi microbial communities were simplified to lactic acid bacteria *Leuconostoc* sp. and *Lactobacillus* sp. over time, with concomitant pH decrease from 6.39 to 4.34 and acidity increase from 0.06% to 0.35%. Other quality characteristics (organic acid, carbon dioxide, and microbial population) also changed, exhibiting high intercorrelation. Pasteurization decreased the initial bacterial counts from 5.20 to 1.92 log CFU/g, thereby delaying the change in quality characteristics (pH, acidity, organic acid, microbial population, carbon dioxide, and microbial community); however, the texture did not differ significantly (*p *< 0.05). In addition, the regression equation for the relationship between acidity and carbon dioxide levels suggested that shelf life could be estimated in conjunction with the ideal gas equation. In conclusion, pasteurization and regression analysis for kimchi shelf life estimation may enable the maintenance of quality and effective management during the distribution process.

## 1 INTRODUCTION

Kimchi, a traditional Korean food, is made by fermenting several vegetables together. Kimchi flavor and functionality depend on the type and quality of the ingredients (kimchi cabbage, salt, red pepper, garlic, ginger, and jeotgal) and fermentation conditions (Ahn, Han, Shin, Jin, & Ghim, 2003), with microorganism‐mediated fermentation and storage temperature constituting important variable factors. Kimchi is recognized as a living food because it produces various physiologically active substances through complicated fermentation processes involving lactic acid bacteria along with various enzymes and microorganisms (Ko, Oh, Oh, & Kim, 2009). Moreover, kimchi has attracted much attention as a health food because its nutritional value and anti‐obesity, atopy improvement, and disease‐preventive characteristics have been scientifically demonstrated (Cui et al., 2015; Lim et al., 2017).

Recently, the demand for commercial kimchi has rapidly increased owing to lifestyle changes. Because kimchi is a fermented food, fermentation progresses during the distribution process and quality changes rapidly, with taste varying depending on fermentation degree. Although the ingestion of overfermented kimchi is not problematic, as kimchi quality is not constant, it is difficult for consumers to select product according to preference or to purchase early‐tasting kimchi. Numerous studies on delayed ripening have attempted to address this problem, including many investigating early‐stage microbial control technologies such as physical (e.g., heat treatment and irradiation), chemical (e.g., preservatives), and biological (e.g., microbial strain inoculation) treatment methods (Jung et al., 2012; Kim et al., 2006). Each carries advantages and disadvantages, with the easiest and most efficient technique being pasteurization. Pasteurization technology is primarily used for foods that undergo quality changes at high temperatures and constitutes a means of reducing microbial load by heating at a low temperature for a short time.

Kimchi is generally distributed in cans, glass bottles, plastic containers, or pouches. However, volume expansion and package leaking and breakage owing to carbon dioxide (CO_2_) generation and pressurization from ongoing fermentation are problematic (Jeong & Yoo, 2016). This shortens kimchi shelf life, resulting in waste and economic loss. Moreover, improved packaging techniques such as CO_2_ permeability control (Lee & Yoo, 2017) and adsorbent usage (Lee, 2016) remain insufficient to fully address these issues. Alternatively, knowledge of kimchi shelf life facilitates product sale and management as it allows anticipation of the returns resulting from packaging damage in advance. Although current practice utilizes CO_2_ control technologies to minimize packaging expansion and breakage, the removal of all CO_2_ in kimchi packaging is not desirable as CO_2_ is a beneficial gas that improves kimchi taste and quality (Lee et al., 2012). To control volume expansion while minimizing CO_2_ loss, it is thus necessary to establish a proper CO_2_ concentration standard in kimchi packaging. For this, prediction of the amount of CO_2_ emissions and establishing a CO_2_ control plan during the distribution process would be required.

The purpose of this study was to investigate the correlation between kimchi fermentation and quality characteristics based on microbial communities. Furthermore, the applicability of pasteurization as a technique to delay lactic acid bacteria growth was examined. To prevent damage caused by packaging breakage during distribution and to respond effectively, the regression analysis of shelf life estimation via the acidity and CO_2_ regression equation was presented.

## 2 MATERIALS AND METHODS

### 2.1 Kimchi preparation and pasteurization

Because each ingredient affects kimchi quality in various ways, in this study, a model kimchi in the form of baik‐kimchi was manufactured by minimizing the factors that could affect the experiment. Ingredients were purchased from a nearby offline market and manufactured as follows. First, kimchi cabbage was cut to 3 × 4 cm size and then pickled in refined salt for 1 hr. Then, garlic (ground using a blender [HR‐1372, Philips, Guangdong, China]) and water were added (kimchi cabbage 90%, refined salt 1.8%, garlic 2.5%, and water 5.7%). Next, 150 g of the prepared kimchi was placed in a pouch (17.5 × 25 × 0.01 cm) and sealed using a sealing machine (AZC‐070, INTRISE, Ansan, South Korea). Pasteurization was performed in a water bath at 55 and 65°C, with sterilization for 30 min. Each sample was stored at 4°C for 4 weeks.

### 2.2 pH, titratable acidity, and salinity determination

Samples were placed in a beaker and the pH was measured using a digital pH electrode (TitroLine Easy, SI Analytics, Mainz, Germany). The titratable acidity was titrated to 10 ml of the filtrate by adding 0.1 N NaOH until pH 8.3, and the consumed 0.1 N NaOH amount was calculated and converted to lactic acid content in %. The salinity was measured by taking 10 ml of 100‐fold diluted kimchi filtrate, adding 2% K_2_CrO_4_, titrating with 0.02 N AgNO_3_ until dark brown, and calculating the consumed amount.

### 2.3 Confirmation of microbial population changes

For analysis of microbial population changes, samples (10 g) were obtained aseptically and diluted 10‐fold with 0.85% NaCl solution in a sterile filter bag, homogenized with a stomacher (Bagmixer R400, Interscience, Saint Nom, France) for 1 min, and then diluted stepwise with 0.85% NaCl solution. Sample dilutions were plated onto plate count agar (Difco, Spark, MD, USA) for total viable bacteria and MRS agar (Lactobacilli MRS agar) for lactic acid bacteria. Colony numbers were then counted by incubating at 30°C for 48 and 72 hr, respectively, and expressed as log CFU/g.

### 2.4 Texture and CO_2_ measurement

Kimchi texture was measured in a one cycle test mode using a CT3 texture analyzer (AMETEK Brookfield, Middleboro, MA, USA) and knife edge probe (TA‐7, 60 mm). The test and posttest speeds were 0.5 and 2 mm/s, the trigger load was 2 g, and after reaching the trigger load, the depth was compressed to 50% of the cabbage surface. Measurements were repeated 10 times per sample, and data were calculated using Texture Pro CT V1.3 software (AMETEK Brookfield). CO_2_ was measured in the headspace and the kimchi juice. Each CO_2_ concentration was measured directly in the packaging using an ISM InPro 5000i CO_2_ sensor (Mettler Toledo, Greifensee, Switzerland).

### 2.5 Microbial community analysis

Total DNA was extracted from kimchi using a PowerSoil DNA Isolation Kit (Cat. No. 12888, MO BIO Laboratories, Carlsbad, CA, USA). DNA concentration and purity were measured using NanoDrop ND 2000 (Thermo Fisher Scientific Inc., Waltham, MA, USA). Polymerase chain reaction (PCR) was performed using primers 16S V3 (5′‐TCG TCG GCA GCG TCA GAT GTG TAT AAG AGA CAG CCT ACG GGN GGC WGC AG‐3′) and 16S V4 (5′‐GTC TCG TGG GCT CGG AGA TGT GTA TAA GAG ACA GGA CTA CHV GGG TAT CTA ATC C‐3′). The PCR protocol was as follows: initial denaturation at 95°C for 2 min, followed by 30 cycles of 95°C denaturation for 20 s, 72°C annealing for 15 s, and 72°C extension for 1 min, and final 72°C extension for 5 min. Sequencing using the Illumina MiSeq platform was performed by Macrogen (Macrogen Inc., Seoul, South Korea). After elimination of sequencing error and ambiguous and chimera sequences using the CD‐HIT Operational Taxonomic Unit (OTU) analysis program, clustering of sequences with similarity ≥97% yielded species‐level OUT. The OTU representative sequence was used to perform UCLUST in the reference database (RDP Release 11, update 4: May 26, 2015) and to generate a taxonomic assignment with the organism information of the subject having the highest homology. QIIME (v1.8) was used to analyze microbial communities.

### 2.6 Organic acid measurement

Organic acids were analyzed by high‐performance liquid chromatography (Ultimate 3000, Thermo Fisher Scientific). Homogenized kimchi was centrifuged (784 x g, 10 min, GRV‐50‐12, VC2200, Labogene, Seoul, South Korea), the supernatant filtered using a 0.2‐μm filter, and the filtrate used for organic acid analysis. Test and standard (lactic acid sodium salt, citric acid, malic acid, succinic acid, oxalic acid, and fumaric acid) solutions (10 μl each) were injected and analyzed using an RI detector (refractive index detector) at a wavelength of 210 nm. The calibration curve was prepared using the standard solution peak, and the organic acid content in the test solution was calculated. The analysis column was used at 40°C with a mobile phase of 0.01 N H_2_SO_4_ solution at 0.5 ml/min.

### 2.7 Correlation, principal component, and regression analyses

The correlation between kimchi quality characteristics and principal component analysis was analyzed using Xlastate software (Xlastate User's Guide, Paris, France). Predicted acidity and CO_2_ regression and regression curves were derived using Sigmaplot 13.0 (SyStat Software, San Jose, CA, USA).

### 2.8 Statistical analysis

Statistical analyses were performed using SPSS ver. 19.0 (Chicago, Il, USA). Two‐way analysis of variance (ANOVA) and Duncan's multiple range comparison tests were employed to determine the level of significance (*p *< 0.05).

## 3 RESULTS AND DISCUSSION

### 3.1 Alteration of kimchi fermentation characteristics by microbial community changes

**Figure 1:**
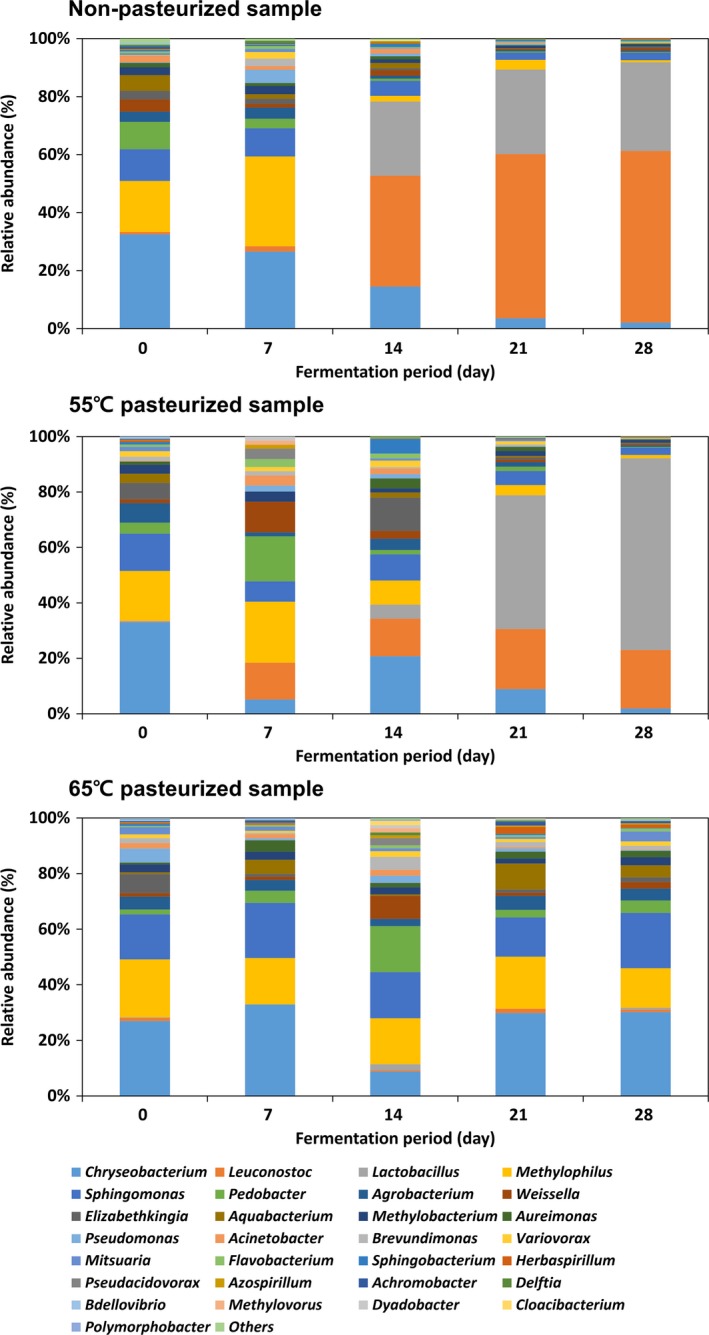
Mean relative abundance of bacterial taxa in kimchi according to pasteurization at the genus level during fermentation. “Others” indicate genera for which the percentage of reads was <0.01% of the total reads in all samples

As the rich taste and flavor of kimchi arise from microorganisms in the fermentation process (Hong, Lee, Kim, & Ahn, 2016), analysis of the types of microorganism growing in kimchi is of practical value. In this study, the 16S rRNA gene was analyzed to investigate microbial community changes in kimchi (Figure 1). At the beginning of fermentation, *Chryseobacterium* sp., *Methyplophilus* sp., *Sphingomonas* sp., and *Pedobacter* sp. which are widely distributed in natural environments accounted for approximately 65%–70% of the dominant species. These microorganisms are salt‐tolerant species derived from ingredients such as kimchi cabbage, garlic, and water. These microorganisms started the initial fermentation.

As kimchi storage time increased, the various microbial communities began to simplify, with *Leuconostoc* sp. and *Lactobacillus* sp. dominating from the 14th fermentation day (Figure 1.). It was considered that the environment was changed by the metabolic activity of various microorganisms within kimchi. In particular, the main factor causing an environmental change was lactic acid bacteria growth, as these bacteria produce various organic acids and CO_2_ by consuming saccharides derived from kimchi cabbage, reducing pH, and increasing acidity to generate an acidic kimchi environment. This environment in turn negatively affects the growth of microorganisms except lactic acid bacteria (Cheigh, 2004; Jung et al., 2011). Moreover, bacteriocin produced by lactic acid bacteria further inhibits the growth of infectious microbes (Han, Lee, Choi, & Paik, 2013). Our findings are consistent with these observations: kimchi pH decreased from 6.39 to 4.34 and acidity increased from 0.06% to 0.35% (Table 1). The salinity range of the kimchi was maintained at 2.42%–2.78% throughout the fermentation process, in contrast to the other features. Headspace and dissolved CO_2_ concentrations continuously increased up to 185.43 and 454.18 mg/L, respectively, with lactic acid bacteria number increasing from 5.10 to 9.13 log CFU/g. Additionally, until the 7th fermentation day, the microbial communities were not simplified to lactic acid bacteria, and pH and acidity were not changed, thus confirming that lactic acid bacteria were the main factor underlying kimchi environment change.

**Table 1:** Physicochemical and microbial characteristics of kimchi according to pasteurization during fermentation

	Fermentation period (day)	Nonpasteurized sample	55°C pasteurized sample	65°C pasteurized sample
pH	0	6.39 ± 0.03^Aa^ †	6.14 ± 0.05^Bb^	5.93 ± 0.04^Ac^
	7	6.43 ± 0.03^Aa^	6.26 ± 0.01^Ab^	5.86 ± 0.01^Bc^
	14	5.22 ± 0.01^Bc^	6.13 ± 0.01^Ba^	5.80 ± 0.03^Cb^
	21	4.63 ± 0.05^Cc^	5.48 ± 0.04^Cb^	5.90 ± 0.03^ABa^
	28	4.34 ± 0.02^Dc^	4.78 ± 0.03^Db^	5.86 ± 0.01^Ba^
Acidity (%)	0	0.06 ± 0.00^Ea^	0.10 ± 0.00^Eb^	0.11 ± 0.00^Dc^
	7	0.08 ± 0.00^Db^	0.11 ± 0.00 ^Da^	0.12 ± 0.00^Ca^
	14	0.17 ± 0.00^Ca^	0.13 ± 0.00^Cc^	0.15 ± 0.00^Ab^
	21	0.24 ± 0.01^Ba^	0.17 ± 0.01^Bb^	0.12 ± 0.01^Cc^
	28	0.35 ± 0.00^Aa^	0.23 ± 0.00^Ab^	0.12 ± 0.00^Bc^
Total viable bacteria (log CFU/g)	0	5.20 ± 0.08^Ea^	3.01 ± 0.01^Eb^	1.92 ± 0.02^Dc^
	7	6.39 ± 0.04 ^Da^	6.27 ± 0.00^Db^	3.15 ± 0.04^Cc^
	14	8.35 ± 0.01^Aa^	7.32 ± 0.03^Cb^	5.05 ± 0.04^Bc^
	21	7.80 ± 0.06^Cb^	8.09 ± 0.01^Ba^	5.74 ± 0.01^Ac^
	28	8.20 ± 0.00^Bb^	8.87 ± 0.09^Aa^	5.64 ± 0.10^Ac^
Lactic acid bacteria (log CFU/g)	0	5.10 ± 0.02^Ea^	3.39 ± 0.12^Eb^	1.96 ± 0.05^Dc^
	7	6.49 ± 0.02 ^Da^	6.38 ± 0.04^Db^	4.17 ± 0.04^Cc^
	14	9.13 ± 0.06^Aa^	7.65 ± 0.04^Cb^	5.12 ± 0.01^Bc^
	21	7.81 ± 0.06^Ca^	8.01 ± 0.10^Ba^	5.63 ± 0.14^Ab^
	28	8.18 ± 0.05^Bb^	8.72 ± 0.08^Aa^	5.47 ± 0.02^Ac^
Hardness (g)	0	6,300.00 ± 441.52^Aa^	6,056.00 ± 353.75^Aa^	5,797.00 ± 601.38^Aa^
	7	5,156.00 ± 250.38^Ba^	5,232.00 ± 217.68^Ba^	5,464.00 ± 207.47^ABa^
	14	4,867.00 ± 386.63^BCa^	4,595.00 ± 210.15^Ca^	4,982.00 ± 260.09^BCa^
	21	4,651.00 ± 416.37^BCa^	4,325.00 ± 429.68^Ca^	4,473.00 ± 410.13^Ca^
	28	4,435.00 ± 671.64^Ca^	3,453.00 ± 237.50^Db^	4,457.00 ± 506.69^Ca^
Headspace carbon dioxide (mg/L)	0	32.85 ± 0.03^Ea^	27.11 ± 0.01^Eb^	22.08 ± 0.02^Ec^
	7	93.18 ± 0.12 ^Da^	56.06 ± 0.04^Db^	33.70 ± 0.01^Dc^
	14	107.79 ± 0.05^Ca^	64.57 ± 0.06^Cb^	38.59 ± 0.04^Cc^
	21	150.85 ± 0.09^Ba^	115.93 ± 0.22^Bb^	43.30 ± 0.03^Bc^
	28	185.43 ± 0.05^Aa^	142.21 ± 0.18^Ab^	48.35 ± 0.69^Ac^
Dissolved carbon dioxide (mg/L)	0	97.30 ± 0.05^Ea^	69.39 ± 0.09^Eb^	57.42 ± 0.02^Ec^
	7	214.23 ± 0.11 ^Da^	138.54 ± 0.09^Db^	69.70 ± 0.04^Dc^
	14	279.35 ± 0.13^Ca^	168.38 ± 0.14^Cb^	76.89 ± 0.06^Cc^
	21	343.98 ± 2.01^Ba^	231.88 ± 0.27^Bb^	104.20 ± 0.04^Bc^
	28	454.18 ± 0.39^Aa^	238.01 ± 0.11^Ab^	111.20 ± 0.07^Ac^

Within columns, values with different uppercase letters are significantly different as per Duncan's multiple range test (*p *< 0.05).

Within rows, values with different lowercase letters are significantly different as per Duncan's multiple range test (*p *< 0.05).

† All values are expressed as the mean ± *SD*.

**Figure 2:**
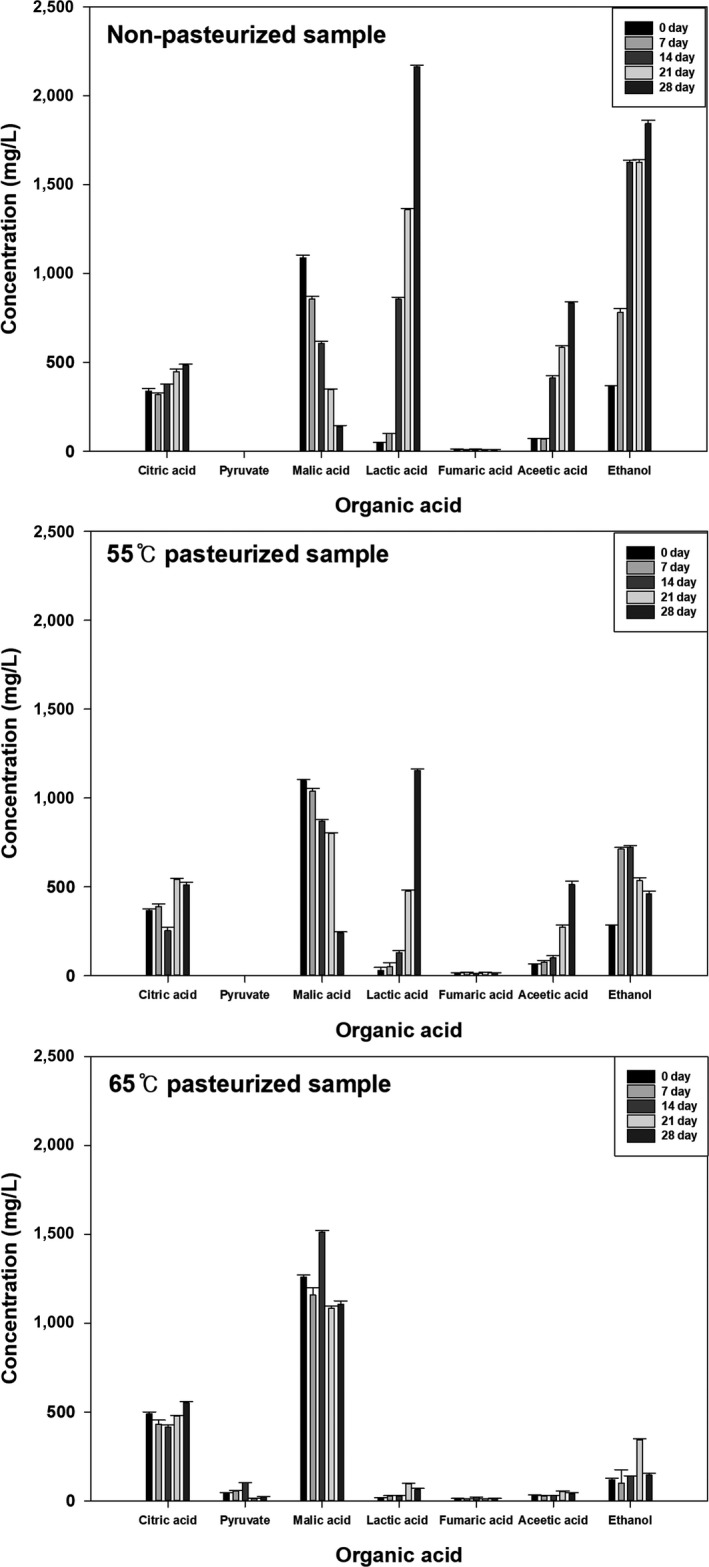
Changes in organic acids of kimchi according to the pasteurization temperature during fermentation

Upon fermentation, various lactic acid bacteria‐produced organic acids are increased or decreased and penetrate into the kimchi and affect the taste (Kim, Kim, Lee, & Noh, 2000). Specifically, *Leuconostoc* sp. and *Lactobacillus* sp. decompose malic acid in kimchi through metabolic processes that break down saccharides and generate organic acids such as acetic and lactic acids, along with CO_2_ (Kim & Lee, 2013). Figure 2 shows the organic acid changes observed during kimchi fermentation in this study. Whereas malic acid decreased, lactic acid, acetic acid, and ethanol increased as fermentation proceeded, which was consistent with previous studies. Lactic and acetic acids are the main substances providing a sour taste and sour flavor in the overfermentation period (Lee, Kim, & Kunz, 2006). Our results thus indicate that the kimchi fermented and had a sour taste and flavor.

### 3.2 Correlation analysis of kimchi quality characteristics

A high correlation was noted between kimchi quality characteristics (Table 2) and lactic acid bacteria growth. As lactic acid bacteria produce organic acids that reduce kimchi juice pH (Jung et al., 2011), the pH, and malic, lactic, and acetic acids were highly intercorrelated. Moreover, as acidity represents lactic acid content in %, the acidity, organic acid content, and pH intercorrelation were also high. The headspace and dissolved CO_2_ concentrations showed a high correlation of 0.969, which is proportional to the partial pressure of the dissolved gas according to Henry's law (Speers & MacIntosh, 2013). The headspace and dissolved CO_2_ intercorrelation with malic, lactic, and acetic acids also exceeded 0.9. As heterofermentative lactic acid bacteria, such as *Leuconostoc* sp., generate CO_2_ and organic acid from oxaloacetate (Cheigh, 2004), a high correlation between these products was also observed.

**Table 2:** Correlation coefficients for quality characteristics in the pasteurized kimchi

Variables	Acidity	pH	Total viable bacteria	Lactic acid bacteria	Headspace carbon dioxide	Dissolve carbon dioxide	Hardness	Citric acid	Pyruvate	Malic acid	Lactic acid	Fumaric acid	Acetic acid	Ethanol
Acidity	1													
pH	−0.956**	1												
Total viable bacteria	0.613*	−0.597*	1											
Lactic acid bacteria	0.606*	−0.595*	0.987**	1										
Headspace carbon dioxide	0.864**	−0.850**	0.831**	0.820**	1									
Dissolve carbon dioxide	0.845**	−0.805**	0.795**	0.795**	0.969**	1								
Hardness	−0.626*	0.627*	−0.758**	−0.727**	−0.659**	−0.535*	1							
Citric acid	0.415	−0.505	0.069	0.004	0.254	0.139	−0.466	1						
Pyruvate	−0.124	0.138	−0.520*	−0.504	−0.451	−0.486	0.163	0.157	1					
Malic acid	−0.793**	0.808**	−0.778**	−0.777**	−0.940**	−0.913**	0.590*	−0.168	0.626*	1				
Lactic acid	0.949**	−0.934**	0.666**	0.660**	0.928**	0.930**	−0.526*	0.303	−0.342	−0.908**	1			
Fumaric acid	−0.191	0.252	−0.182	−0.209	−0.355	−0.463	0.014	0.171	0.372	0.454	−0.377	1		
Acetic acid	0.934**	−0.932**	0.704**	0.700**	0.941**	0.937**	−0.532*	0.285	−0.390	−0.928**	0.995**	−0.353	1	
Ethanol	0.694**	−0.661**	0.674**	0.707**	0.805**	0.912**	−0.284	−0.113	−0.492	−0.786**	0.812**	−0.546*	0.824**	1

Significant at *p < 0.05 and **p < 0.01.

### 3.3 Pasteurization effects on kimchi fermentation characteristics

The observed correlations suggested that if lactic acid bacteria growth was delayed, the quality change would be slow. We, therefore, assessed the effects of pasteurization on microbial community and fermentation characteristics (Figures 1 and 2, Table 1). At the beginning of fermentation, similar microbial communities were identified in both the nonpasteurized and pasteurized samples at 55 and 65°C, although pasteurization reduced the initial microorganism numbers. *Leuconostoc* sp. and *Lactobacillus* sp. were the dominant species on the 14th day after no pasteurization and 21^st^ day after pasteurization day at 55°C, whereas lactic acid bacteria growth was not confirmed upon 65°C pasteurization. Lactic acid bacteria growth rate differences affected kimchi quality. Although the organic acid increase and decrease patterns were the same for 55°C pasteurized and nonpasteurized samples, the amount of increase or decrease differed depending on the lactic acid bacteria growth rate. Organic acid changes could not be confirmed in the pasteurized samples at 65°C, which was possibly due to the lack of active lactic acid bacteria growth. Moreover, the rate of change in pH, acidity, and CO_2_ was dependent on the pasteurization temperature; as the pasteurization temperature increased, kimchi ripening, as determined by quality characteristics, was delayed.

As heating sterilization may cause soft texture, use of a heat level that does not cause deterioration is important. Nonpasteurized, 55°C, and 65°C pasteurized samples showed values of 6,300, 6,056, and 5,797 g, respectively, after treatment (Table 1), and the difference was not significant (*p *< 0.05). However, texture decreased because of softening as storage time passed. Such softening occurs because the α ‐1,4 bond of polygalacturonic acid, which is the basic structural component of pectin, is hydrolyzed by polymethylgalacturonase and polygalacturonase, which degrade the internal structure of kimchi cabbage and comprise microbial secretory enzymes in kimchi (Park, Kim, & Oh, 2016). Previously reported studies indicated that pasteurization at 65°C for 30 min was the most efficient and that brined kimchi cabbage also had the effect of prolonging the storage period with pasteurization at 65°C for 30 min (Lee, 2010). These pasteurization conditions prolonged the storage period for brined cabbage in the study by Lee (2010) and in the present study.

**Figure 3:**
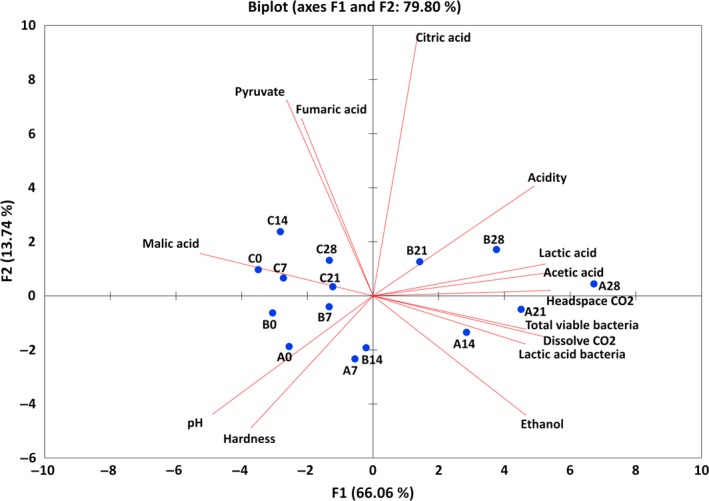
Principal component analysis of the physicochemical and microbial properties of kimchi according to pasteurization (A, B, and C = non‐, 55°C, and 65°C pasteurized sample, respectively)

Figure 3 also illustrates that pasteurization delayed kimchi fermentation, as determined through principal component analysis. Headspace and dissolved CO_2_, acidity, total viable bacteria, acetic acid, lactic acid, and ethanol were located in the positive direction of F1, whereas pH, malic acid, hardness, pyruvate, and fumaric acid were located in the F1 negative direction. The quality characteristics that were generated or increased through the fermentation process were in the positive F1 direction, whereas those appearing to decrease or disappear were in the negative direction. The spot of each sample shown in Figure 3 demonstrates a tendency to move from left to right in the F1 phase with increase in storage time. The combination of the quality characteristic location and the sample spot suggested that the fermentation proceeded from left to right on F1. The rate of movement to the right was highest in the nonpasteurized sample, followed by the 55°C pasteurized sample. The 65°C pasteurized sample did not appear to progress to fermentation in terms of microbial community, pH, and acidity, whereas it moved slowly to the right at the negative F1 position, suggesting very slow fermentation progress. Overall, the principal component analysis confirmed that ripening was delayed when the initial microbial population was reduced by pasteurization.

### 3.4 Regression analysis for kimchi shelf life estimation

**Figure 4:**
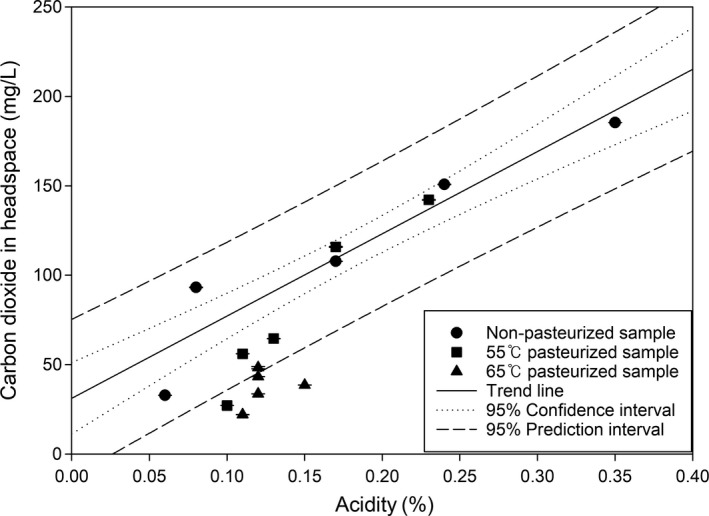
Regression analysis of acidity and carbon dioxide on kimchi according to pasteurization

CO_2_ plays a beneficial role in kimchi fermentation, although excessive CO_2_ generation causes volume expansion of kimchi packaging, shortening the shelf life. Optimal CO_2_ control is, therefore, required; however, there is no clear understanding as to what degree of CO_2_ control is required. Accordingly, it was considered that the regression analysis of kimchi CO_2_ emission and the optimal CO_2_ concentration in the packaging would be useful for kimchi distribution and quality control. For regression analysis of kimchi CO_2_ emission, the experimental results were statistically plotted as a regression line between acidity and CO_2_ (Figure 4). The equation for the regression line is shown in the following Equation (1).

(1) $$Y=460.2842105263X+31.1688421053,\;r^{2}=0.89$$

The coefficients of determination in the regression equation showed a high value of 0.89, indicating that the predicted regression lines showed high intervariable reliability. When the acidity and CO_2_ values of pasteurized samples were substituted in (1), the 55°C pasteurized sample was included in the prediction interval, and it was confirmed that the amount of CO_2_ emission relative to the acidity could be predicted by substituting any sample in (1).

As the standards for determining the optimum CO_2_ concentration level in kimchi packaging may vary from person to person, the CO_2_ concentration accumulated up to the time of packaging expansion or breakage was considered when establishing a clear standard. The standard was set using the ideal gas Equation (2) (Lee, Shin, Lee, Kim, & Cheigh, 2001).

(2) $$C_{{\mathrm{CO}}_{2}}=100,000\times P_{{\mathrm{CO}}_{2}}\times M_{{\mathrm{CO}}_{2}}/\mathrm{RT}$$

where $C_{{\mathrm{CO}}_{2}}$ is the CO_2_ concentration in the headspace (mg/ml), $P_{{\mathrm{CO}}_{2}}$ is the partial pressure of CO_2_ in the headspace (bar), $M_{{\mathrm{CO}}_{2}}$ is the molar mass of CO_2_ (0.044 kg/mol), *R* is the gas constant (8.314 J/K·mol), and T is the temperature in Kelvin.

For simplicity, the various types of kimchi packaging were classified as rigid and flexible. For rigid containers, the volume does not change with CO_2_ generation although the internal pressure changes; therefore, the pressure at the time of breakage or leakage owing to increased internal pressure was set as a standard. For flexible packaging, the volume rather than the pressure changes; therefore, the pressure at the time when the internal pressure of the flexible packaging becomes higher than the atmospheric pressure was set as a standard. The maximum headspace CO_2_ concentration in the package can be predicted by substituting the standard pressure value of each type of packaging into the right side of Equation (2).

To predict packaging breakage time during kimchi distribution, the acidity of the packaging breaking point should be deduced through Equations (1) and (2). The CO_2_ concentration in the headspace is predicted through Equation (2), and the X (acidity) value can be derived by substituting the predicted value into the Y variable in Equation (1). As acidity is a quality index that predicts the degree of kimchi fermentation, it is possible to predict shelf life according to the storage temperature. A model for estimating the CO_2_ emissions by acidity has already been proposed by Lee, Kwon, and Ha (1997), which predicted the CO_2_ emissions from theoretical equations and experimental data. However, this modeling is complicated and difficult to formulate, especially for application by nonexperts, as the application standards differ according to the effect stage. In contrast, the regression analysis proposed in this study should be more efficient because the calculation process is simple, and thus, the model is more practical for use in the industry.

## 4 CONCLUSIONS

Investigation of changes in kimchi fermentation characteristics indicated high intercorrelation among quality characteristics. The fermentation‐retarding effect was confirmed according to quality (pH, acidity, organic acid, microbial population, and CO_2_) and principal component analyses. Notably, pasteurization caused no significant difference in texture between the samples. It is expected that the regression analysis developed for shelf life estimation of kimchi would be easy to use in the field to effectively manage the problems of kimchi packaging volume expansion and breakage.

## CONFLICT OF INTEREST

The authors declare that they do not have any conflict of interest.

## ETHICAL STATEMENT

This study does not involve any human or animal testing.
